# Towards a European health research and innovation cloud (HRIC)

**DOI:** 10.1186/s13073-020-0713-z

**Published:** 2020-02-19

**Authors:** F. M. Aarestrup, A. Albeyatti, W. J. Armitage, C. Auffray, L. Augello, R. Balling, N. Benhabiles, G. Bertolini, J. G. Bjaalie, M. Black, N. Blomberg, P. Bogaert, M. Bubak, B. Claerhout, L. Clarke, B. De Meulder, G. D’Errico, A. Di Meglio, N. Forgo, C. Gans-Combe, A. E. Gray, I. Gut, A. Gyllenberg, G. Hemmrich-Stanisak, L. Hjorth, Y. Ioannidis, S. Jarmalaite, A. Kel, F. Kherif, J. O. Korbel, C. Larue, M. Laszlo, A. Maas, L. Magalhaes, I. Manneh-Vangramberen, E. Morley-Fletcher, C. Ohmann, P. Oksvold, N. P. Oxtoby, I. Perseil, V. Pezoulas, O. Riess, H. Riper, J. Roca, P. Rosenstiel, P. Sabatier, F. Sanz, M. Tayeb, G. Thomassen, J. Van Bussel, M. Van den Bulcke, H. Van Oyen

**Affiliations:** 1 Technical University of Denmark, Kongens Lyngby, Denmark; 2 Medicalchain, York Road, London, SQ1 7NQ UK; 3 National Health Service, London, UK; 4 Translation Health Sciences, Bristol Medical School, Bristol, BS81UD UK; 5 European Institute for Systems Biology and Medicine (EISBM), Vourles, France; 6 Regional Agency for Innovation & Procurement (ARIA), Welfare Services Division, Lombardy, Milan, Italy; 7 Luxembourg Centre for Systems Biomedicine, Campus Belval, University of Luxembourg, Luxembourg City, Luxembourg; 8 CEA, French Atomic Energy and Alternative Energy Commission, Direction de la Recherche Fondamentale, Université Paris-Saclay, F-91191 Gif-sur-Yvette, France; 9 Istituto di Ricerche Farmacologiche Mario Negri IRCCS, Bergamo, Italy; 10 Institute of Basic Medical Sciences, University of Oslo, Oslo, Norway; 11 Ulster University, Belfast, BT15 1ED UK; 12 ELIXIR, Welcome Genome Campus, Hinxton, Cambridge, CB10 1SD UK; 13 Sciensano, Brussels, Belgium and Tilburg University, Tilburg, The Netherlands; 14 Department of Computer Science and Academic Computing Center Cyfronet, Akademia Gornizco Hutnizca University of Science and Technology, Krakow, Poland; 15 Ghent University, Ghent, Belgium; 16 European Molecular Biology Laboratory, European Bioinformatics Institute, Wellcome Genome Campus, Hinxton, Cambridge, CB10 1SD UK; 17 Fondazione Toscana Life Sciences, 53100 Siena, Italy; 18 CERN, European Organization for Nuclear Research, Meyrin, Switzerland; 19 University of Vienna, Vienna, Austria; 20 INSEEC School of Business & Economics, Paris, France; 21 PwC, Dronning Eufemiasgate, N-0191 Oslo, Norway; 22 Center for Genomic Regulations, Barcelona, Spain; 23 Neuroimmunology Unit, The Karolinska Neuroimmunology & Multiple Sclerosis Centre, Department of Clinical Neuroscience, Karolinska Institute, Stockholm, Sweden; 24 Institute of Clinical Molecular Biology, Kiel University and University Hospital Schleswig-Holstein, Campus Kiel, Kiel, Germany; 25 Department of Clinical Sciences, Pediatrics, Lund University, Skåne University Hospital, Lund, Sweden; 26 Athena Research & Innovation Center and University of Athens, Athens, Greece; 27 National Cancer Institute, Vilnius, Lithuania; 28 geneXplain GmbH, Wolfenbüttel, Germany; 29 Centre Hospitalier Universitaire Vaudois, Lausanne, Switzerland; 30 European Molecular Biology Laboratory, Genome Biology Unit, Heidelberg, Germany; 31 Integrated Biobank of Luxembourg, Rue Louis Rech, L-3555 Dudelange, Luxembourg; 32 https://www.mitzilaszlo.org; 33 Antwerp University Hospital and University of Antwerp, Edegem, Belgium; 34 Clinerion Ltd, Elisabethenanlage, 4051 Basel, Switzerland; 35 European Cancer Patient Coalition, Rue de Montoyer/Montoyerstraat, B-1000 Brussels, Belgium; 36 Lynkeus, Via Livenza, 00198 Rome, Italy; 37 Public Policy Consultant, Rome, Italy; 38 European Clinical Research Infrastructure Network, Heinrich-Heine-Universität, Düsseldorf, Germany; 39 Science for Life Laboratory, KTH Royal Institute of Technology, Stockholm, Sweden; 40 Centre for Medical Image Computing, Department of Computer Science, University College London, London, UK; 41 Information Technology Department, Institut National de la Santé et de la Recherche Médicale, Paris, France; 42 Unit of Medical Technology and Intelligent Information Systems, Department of Materials Science and Engineering, University of Ioannina, Ioannina, Greece; 43 Institute of Medical Genetics and Applied Genomics, Rare Disease Center, Tübingen, Germany; 44 Section Clinical, Neuro and Developmental Psychology, Department of Behavioural and Movement Sciences, Vrije Universiteit, Amsterdam, The Netherlands; 45 Hospital Clínic de Barcelona, IDIBAPS, University of Barcelona, Barcelona, Spain; 46 French National Centre for Scientific Research, Grenoble, France; 47 Hospital del Mar Medical Research Institute (IMIM), Universitat Pompeu Fabra, Barcelona, Spain; 48 University of Oslo, Oslo, Norway; 49 Scientific Institute of Public Health, Brussels, Belgium; 50 Sciensano, Juliette Wystmanstraat, 1050 Brussels, Belgium

## Abstract

The European Union (EU) initiative on the Digital Transformation of Health and Care (Digicare) aims to provide the conditions necessary for building a secure, flexible, and decentralized digital health infrastructure. Creating a European Health Research and Innovation Cloud (HRIC) within this environment should enable data sharing and analysis for health research across the EU, in compliance with data protection legislation while preserving the full trust of the participants. Such a HRIC should learn from and build on existing data infrastructures, integrate best practices, and focus on the concrete needs of the community in terms of technologies, governance, management, regulation, and ethics requirements. Here, we describe the vision and expected benefits of digital data sharing in health research activities and present a roadmap that fosters the opportunities while answering the challenges of implementing a HRIC. For this, we put forward five specific recommendations and action points to ensure that a European HRIC: i) is built on established standards and guidelines, providing cloud technologies through an open and decentralized infrastructure; ii) is developed and certified to the highest standards of interoperability and data security that can be trusted by all stakeholders; iii) is supported by a robust ethical and legal framework that is compliant with the EU General Data Protection Regulation (GDPR); iv) establishes a proper environment for the training of new generations of data and medical scientists; and v) stimulates research and innovation in transnational collaborations through public and private initiatives and partnerships funded by the EU through Horizon 2020 and Horizon Europe.

## Background

Genomics has brought life sciences into the realm of data sciences—large-scale DNA and RNA sequencing is now routine in life-science and biomedical research, with an estimate of up to 60 million human genomes available in the coming years [1, 2]. Recent innovations in medical research and healthcare, such as high-throughput genome sequencing, transcriptomics, proteomics, metabolomics, single-cell omics techniques, high-resolution imaging, electronic health and medical records (EHRs/EMRs), big-data analytics, and a plethora of internet-connected health devices, fundamentally change the infrastructure requirements for health research.

Translating these new data together with clinical information into scientific insights and actionable outcomes for improving clinical care is a major challenge. As life-science and health research datasets rapidly grow larger, with an ever-increasing number of study participants required to detect meaningful but weak signals that may be blurred by a myriad of confounding biological, experimental, or environmental factors, the computational resources required to process and analyze this big data increasingly outgrows the capabilities of even large research institutes. The various cloud technologies and services defined in Table 1 are based on shared commercial and private computer and storage resources that can be provided on demand to users from a large number of different institutions who are conducting or participating in joint projects. They have emerged as powerful solutions to the challenges of collaborating in research on genomic, biomedical, and health data.

**Table 1:** Glossary of cloud computing terms

Application	A set of programs running on one or more computers allowing a user to perform a set of tasks
Cloud application	An application running in the cloud
Cloud computing	The delivery of IT services over a network (e.g., the internet) by means of a combination of infrastructure, software, and data hosted by one or more cloud providers using a service model similar to that used by traditional utility companies (e.g., water or electricity)
Cloud federation	The combination of infrastructure, software, and services from separate networks and providers, having shared access mechanisms, to perform common actions, achieve load-balancing or optimize availability or costs
Cloud instance	A virtual server or container of resources running on a physical host computer possibly hosting several independent instances (see virtualization)
Cloud marketplace	An online marketplace of cloud services and applications operated by a Cloud Service Provider (CSP)
Cloud service provider (CSP)	A company or public entity that offers cloud services to individual users or other entities
Cloud storage	A model of data storage in which data are hosted across one or more facilities by a hosting entity or CSP and remotely accessed by users over the internet
Container	A type of virtualized instance running on a physical host server in isolated user spaces and possibly preloaded with applications
Hybrid cloud	A cloud computing infrastructure comprised of a mix of public and private cloud and on-premise instances and resources
Infrastructure	The combination of hardware resources (network, computing, storage, etc.) and virtualized instances supporting an IT environment
Infrastructure as a Service (IaaS)	A model of cloud computing in which a CSP provides an infrastructure of virtualized resources to users as a service over a network (e.g., the internet)
On-premise	Referring to infrastructure or software that is run on computing resources that are physically hosted by the entity using them
Platform	A computer system on which applications can run on or can be built
Platform as a Service (PaaS)	A model of cloud computing in which a CSP provides the infrastructure and the platforms where users can run and manage their own applications as a service over a network (e.g., the internet)
Private cloud	A cloud infrastructure used by a single organization either on-premise or hosted by a third-party CSP over the internet or dedicated private networks
Public cloud	A cloud infrastructure hosted by a CSP (or a federation) and used by the public or multiple organizations across the internet
Software as a Service (SaaS)	A model of cloud computing in which a CSP hosts and provides applications (software) to users as a service over a network (e.g., the internet)
Virtualization	A technology that allows users to run a software simulation of a physical computer on which a full operating system and applications can be installed

Biomedical and health research has yet to enter fully the big data and cloud computing era. The Health Research and Innovation Cloud (HRIC), as described in this manuscript, would help to facilitate this transition, providing access to larger datasets, cutting-edge tools, and knowledge, as envisioned by Auffray et al. [3]. For example, the HRIC should ease the incorporation of domain expert knowledge into systems disease maps in a format that can be both understood by all stakeholders (patients and clinicians, scientists, and drug developers) and processed by high-performance computers, thus supporting the development of innovative medicines and diagnostics [4, 5]. Cloud technologies (accessed through Hadoop applications, for example) also make it possible to collaborate and to access and reuse data in situations when privacy concerns or regulation prohibits remote users from downloading data—an important benefit in Europe where national regulations can differ significantly. Clouds allow algorithms to be brought to the data, and as such can enable data sharing and joint processing without generating unnecessary copies of the data, which comes with potential benefits for data protection [6, 7]. In addition, clouds make it possible to perform computational analyses at a scale that individual institutions would struggle to manage [7]. Consequently, in the past few years, the large international cancer and other genomics consortia have created specialized genomics and biomedical cloud environments, each supporting individual projects [2]. These projects have made important advances in connecting health research data across disciplines, organizations, and national boundaries. For instance, in research on rare diseases, international collaborations that integrate genomic, phenotypic, and clinical data have introduced new paradigms in diagnosis and care [8]. However, a project-based, fragmented landscape will not enable access to and construction of the large data cohorts that are required to address novel or broader biomedical questions that were not anticipated when collecting informed consent from participants in individual projects, nor will it provide adequate data governance and containable cost models.

Scaling and sustainably managing such solutions to support all European life scientists thus require a coordinated action from science policy makers, funders, and other actors in this complex ecosystem. Connecting Europe’s health data to advance the understanding of life and disease requires that research data and analysis tools, standards, and computational services are made FAIR—that is, findable, accessible, interoperable, and reusable—for researchers across scientific disciplines and national boundaries [9]. Truly enabling personalized and digital medicine across Europe and beyond will require a connected digital infrastructure for Europe’s health data that supports systematic openness and the integration of research data with real-world datasets (e.g., environmental monitoring data) generated inside all of the healthcare systems, government agencies, foundations, and private organizations that will adopt it.

On 13 March 2018, the Health directorate of the Directorate-General for Research and Innovation of the European Commission of the EU organized a workshop to explore the possibility of and challenges involved in establishing a cloud for health research and innovation, which would be accessible by researchers and health professionals throughout Europe, in line with recommendations for a European Innovation Council and the Horizon Europe 2021–2027 framework program [10, 11]. The cloud-computing environment proposed in this manuscript builds upon the European Open Science Cloud (EOSC) initiative developed in the past few years by the European Commission [12], with a focus in the life science and medicine fields. The EOSC aims at developing a trusted, open environment in which the scientific community can store, share, and re-use scientific data and results. Overall, the authors feel that the cloud described in this manuscript, which would provide the biomedical and health research community with the technical infrastructure and services necessary to support the development of innovative diagnostics methods and medical treatments, should become an integral part of the EOSC. The workshop gathered a broad range of experts from multiple biomedical research disciplines, health care, informatics, ethics, and legislation, including representatives of more than 45 collaborative projects funded by the EU through the FP7 (The European Union’s seventh framework programme for Research, Technological Development and Demonstration) and Horizon 2020 (H2020) programs. The participants explored requirements and developed a set of recommendations for a European HRIC to connect researchers and health data sources in Europe [13]. The main aim of the HRIC is for clinical data, software, computational resources, methods, clinical protocols, and publications to be more widely and securely accessed and reused following the FAIR principles [9] than is currently possible with existing European research infrastructures, such as ELIXIR, that form a network of heterogeneous national nodes. For example, the HRIC infrastructure would benefit from the aforementioned advantages of cloud computing in the archiving and dissemination of health data.

This paper summarizes the main conclusions from the workshop and highlights five recommendations and action points to the EU and national stakeholders (Table 2). The recommendations are key issues that need to be addressed in order to link biological, clinical, environmental, and lifestyle information (from single individuals to large cohorts) to the health and wellbeing status of patients and citizens over time, while making this wealth of data and information available for European health research and innovation in clinical care.

**Table 2:** Summary of the recommendations, details on the rationale, and suggestions for action points directed to the funding agencies and the actors in the field

Recommendation	Rationale	Action points
Provide and foster standards, good practices, and guidelines necessary to establish the European Health Research and Innovation Cloud (HRIC)	The HRIC should be supported by predefined standards, data formats, protocols, and templates. The data standards and guidelines applied in the HRIC should be designed to facilitate interoperability between the diverse health systems and policies in Europe and globally	Suggest the adoption of data formats and architectures in policies, grant applications, and projects calls throughout the EU and its member states
Develop and certify the infrastructure and services required for operation of the HRIC	The HRIC should provide computational infrastructures and services and analytical and visualization tools to all users as a platform to share knowledge, data, and guidelines. Services for data sharing, security, and analysis should be compliant with an EU certification system	- In future grant and project calls related to the development of the HRIC, the EU and the applicants should commit to complying with the highest standards of security, interoperability, and reproducibility - The EU should develop a certification system to validate compliance with the standards mentioned
Enable the HRIC to operate within an ethical and legal framework that is adequate for health systems	A robust ethical and legal framework has to be developed that defines rules for privacy, security, ownership, access, and usage of data within the HRIC. A federated system architecture should be preferred as it allows for comparison of data and results, while complying with EU General Data Protection Regulation (GDPR) and international data protection and sharing rules	- In grants and project calls, federated and GDPR-compliant data architectures should be preferred
Establish a proper environment for the training of a new generation of data and medical scientists	Education and training of health professionals need to be updated with the HRIC in mind, considering both international standards and practices for data sharing as well as national environments and regulations. The EU should take inspiration in existing large and successful infrastructures that foster multidisciplinary teams, such as the European Organisation for Nuclear Research (CERN)	- Scientists and health professionals in training should be made aware of the possibilities of the HRIC - Communication in relevant professional channels should be strengthened
Fund public and private initiatives for the development of the HRIC through EU Framework Programmes (Horizon 2020 and Horizon Europe)	The EU and its member states should, together with private investors, develop a coherent, ambitious, and long-term action plan supported by innovative funding mechanisms that consolidate the outcomes from the existing project portfolio into a long-term operational infrastructure	- The EU should invest through calls and grants in order to build and consolidate the HRIC - The existing industrial ecosystem should be supported, to remain competitive against the other actors in the world

## The HRIC should be built on established standards and guidelines, to foster European-wide medical research

### Rationale

Sharing of data, information, and knowledge represents the most important functionality in the context of a HRIC. High-level standardization, common exchange mechanisms, interfaces and protocols, and semantic interoperability form the foundation for widespread adoption of the FAIR principles [9] in health research. Data that are shared collaboratively in such health-related cloud projects are now largely standardized for the processing of genomic DNA read and genomic variant-calling files. By comparison, the sharing of highly sensitive clinical and health data has been much less developed to date and hence represents a key area for future focus. Numerous challenges remain with regard to sharing these data in a meaningful way.

### Existing standards and guidelines

Many individual projects, in Europe and globally, have demonstrated the opportunities and the added value presented by connecting and exchanging data across countries via standardized protocols. Table 3 lists recent European projects that have developed towards the exchange of clinical and health data using cloud-based solutions. All of those projects have developed and implemented worthy ideas that should be included in the HRIC. However, we envision the HRIC as a disease-agnostic environment, and on a larger scale than the platforms mentioned in Table 3. Moreover, the HRIC should not be tied to a single project or consortium, but should rather be under the governance of an independent body. International exchange of health research data holds tremendous potential in disease research by facilitating better investigation of disease causality and linking of genotypes and phenotypes, as has been demonstrated, for example, in the Pan-Cancer Analysis of Whole Genomes (PCAWG) project. An important aspect of cloud-based data governance is that it allows sharing of data outside a consortium through a data request mechanism and a governance infrastructure that track participant consent and data access. The cloud-based audit trail capabilities of cloud-based research analyses, which can be implemented at both data and infrastructure levels, is a direct benefit to the data controllers. A standardized data model (or data access model) and/or standardized metadata models facilitate the consolidation of different data sets and significantly increase the findability, the semantic interoperability, and, as a consequence, the reusability of data, and thus its ‘FAIRness’ [9, 14].

**Table 3:** Relevant initiatives for the European Health Research and Innovation Cloud (HRIC)

Project	Aims/summary	Cloud model used	References
CORBEL project	Creating a platform for harmonized user access to biological and medical technologies, biological samples, and data. The project has developed the data harmonization, ethics guidance, and user-access protocols necessary for transnational access to both pre-clinical and clinical research infrastructures and is piloting access to participant-level data from clinical trials [43]	Scalable cloud-based provision of data access and compute across infrastructures	[59]
ELIXIR	European research infrastructure with 21 members and over 180 research organizations. ELIXIR is creating a network of local instances of the European Genome-Phenome Archive that give users controlled and secure access to raw data and precomputed results [44, 45]	Hybrid cloud ecosystem: i) Local, private clouds (e.g., EMBL-EBI Embassy) ii) National community clouds (e.g., cPouta, MetaCentrum cloud, de. NBI) iii) European research and innovation-oriented clouds (e.g., European Open Science Cloud (EOSC)) iv) Public/commercial compliant clouds (e.g., Google, Azure, Amazon web Service (AWS))	[60]
European Translational Information and Knowledge Management Services (eTRIKS)	IMI-funded highly scalable cloud-based platform for translational research, information, and knowledge management providing open-source applications that can securely host heterogeneous data types, including multi-omics data, preclinical laboratory data, and clinical information, including longitudinal data sets. The platform is a robust translational research knowledge management system that is able to host other data-mining applications and support the development of new analytical tools [46]	Scalable cloud-based platform for translational research and applications development. The Openstack technology is used to run a private cloud for eTRIKS	[61]
European Medical Information Framework (EMIF)	Innovative Medicines Initiative (IMI)-funded project that has successfully improved access to human health data by providing tools and workflows that can be used to discover, assess, access, and (re) use human health data. The efforts of this IMI project are being extended through the European Health Data and Evidence Network (EHDEN) project	Research analytical service approaches from EHR and cohort data platforms	[62, 63]
Human Brain Project (HBP) Medical Informatics Platform	The HBP Medical Informatics Platform allows researchers around the world to exploit medical data to create machine-learning tools that can analyze these data for new insights into brain-related diseases. The Medical Informatics web-portal [47] provides a software framework, based on federated and distributed computing, that allows researchers to mine clinical data stored on hospital and laboratory servers, without moving the data from the servers where they reside and without compromising patient privacy	The HBP Joint Platform plans to adopt cloud technology and provide those services through its computer centers (JSC-Jülich and CSCS-Lugano) together with BSC-Barcelona, CINECA-Bologna, and CEA-Saclay. Software infrastructure: i) The (base) infrastructure layer is accessible through an ‘Infrastructure as a Service’ (IaaS) interface ii) Tools to enable simulation and modeling as well as data analytics workflows for neuroscience, which the HBP operates as a “Platform as a Service” (PaaS) iii) Several software services for data-driven brain simulations and for virtual neurorobot design and operation offered in the form of ‘Software as a Service’ (SaaS). HBP operates the following SaaS: a) Model-driven brain simulations b) Neurorobotics simulation and development tools across the whole workflow the of neurorobotics life cycle	[64]
Human Brain Project (HBP) Knowledge Graph Data Platform	The HPB Knowledge Graph (KG) is an online graph database that accepts submissions of anonymized human data, animal data, and models from the brain. All data that are made discoverable and accessible through a KG search have been curated. Data are also made available with integrated multilevel HBP Atlases, holding information about the brain in standard reference spaces		[65]
Helix Nebula	A pan-European public–private partnership initiative led by EIROforum and leading commercial cloud-computing partners. Since 2011, this project has been piloting the use of cloud computing to enable complex data analyses and large-scale data sharing, with life science-oriented projects ranging from complex genome assembly to assessing somatic variation in the context of different types of cancer	Helix Nebula Science Cloud (HNSciCloud): hybrid cloud platform that links together commercial cloud service providers and publicly funded research organizations’ in-house IT resources via the GEANT network to provide innovative solutions supporting data intensive science. These services support the connection of the research infrastructures identified in the European Strategy Forum on Research Infrastructures (ESFRI) Roadmap to the nascent European Open Science Cloud (EOSC) and are intended to create a single digital research space for Europe’s 1.8 million researchers	[66]
European Open Science Cloud (EOSC)	The pilot project has recently investigated the benefits of data and cloud computer sharing at a pan-European level in life science-oriented projects on pan-cancer analyses [31] and imaging. This initiative has been pursued across academic cloud-computing environments located in Western and Eastern Europe as well as Canada. Similar initiatives have been launched in the USA [8]	Cloud-based services for open sciences—integration and consolidation of e-infrastructure platforms, federation of existing European research infrastructures and scientific clouds	[25]
Pancancer Analysis of Whole Genomes (PCAWG)	An international collaboration to identify common patterns of mutation in more than 2800 cancer whole genomes from the International Cancer Genome Consortium. This project is exploring the nature and consequences of somatic and germline variations in both coding and non-coding regions, with specific emphasis on cis-regulatory sites, non-coding RNAs, and large-scale structural alterations	Hybrid cloud model. The data-coordinating center lists collaborative agreements with cloud providers’ AWS and an academic computing cloud resource maintained at the cancer collaboratory, by the Ontario Institute for Cancer Research and hosted at the Compute Canada facility	[67, 68]
RD-Connect	RD-Connect is an integrated platform connecting databases, patient registry data, biobanks, and clinical bioinformatics for rare disease research. It allows the integration of different data types (e.g., omics, clinical information, patient registries, and biobanks). Those integrated data can be accessed and analyzed by the scientific community to speed up research, diagnosis, and therapy development for patients with rare diseases	Online secured platform connecting different types of patient-related rare disease data, enabling genome-phenome analysis	[69]
COMPARE	A network of collaborators of the Global Microbial Identifier initiative (GMI) that aims to improve the identification and mitigation of emerging infectious diseases and foodborne outbreaks	One-serve-all analytical framework and data exchange platform with various data integration for real-time analysis and interpretation of pathogen sequence data	[70, 71]

Workshop participants agreed that a first minimalistic and yet effective approach to data exchange should consist of a small number of initial online repositories containing references (e.g., links to render data sets findable) along with metadata (e.g., type and scale of content, specifications describing in what systems the data set may be stored and processed), and indications on how to gain access (e.g., requirements and point of contact). This could be designed as a metadata repository containing the metadata of data objects and information on how to gain access to them. The data objects as such may, or indeed should, be stored elsewhere.

Beyond their metadata, however, data sets are bound to differ greatly because research projects vary largely in scope. Not only would it be cumbersome to record a large number of parameters that are irrelevant to the specific question addressed, but it would also be problematic from an ethics viewpoint, considering patient personal data protection aspects [15]. It therefore seems more promising to drive standardization within research communities while looking out for opportunities for overall standardization.

Thus, the workshop envisioned the HRIC as a distributed collection of data repositories, people, and services, which together make up a framework for sharing and operating as a federated data commons, with reproducible software, standards, and expertise based on joint policies and guidelines on conducting health research, much like the smaller frameworks used successfully in previous initiatives [2, 16–19]. The need for federation is also highlighted in the proposed EU action plan for ‘Making sense of big data in health research’ [3]. Creating such a HRIC opens new frontiers for research and healthcare via the opportunities for strong international collaborations.

Beyond Europe, the HRIC should collaborate internationally to drive the development and widespread adoption of global standards and connectivity. Ongoing initiatives exist outside Europe that are aiming to develop global standards for the secure exchange of data sets such as health and medical records within health information federations, while tracking the completeness of the supporting data [20, 21]. These initiatives also aim to develop guidelines for data analytics and standardized workflows. They should be considered for the basis of the HRIC as this will provide the scientific community with means of reproducibility, version control, and documentation, which will be an important vehicle to drive increased standardization and connectivity.

## A European HRIC should be developed and certified to the highest standards of interoperability and data security

### Rationale

The workshop participants enthusiastically endorsed the vision of the HRIC as a federated environment. The major blockers for cross-border collaborations in translational research on disease prevention, treatment, and management will be addressed through a federated HRIC infrastructure with work on the standardization, harmonization, and integration of genomic data with other health-relevant information to optimize hypothesis-driven analyses. The data sources will remain at their location of origin and are made accessible to users through a metadata repository. Moreover, data security has to be integral to the development of the HRIC, and modern cryptology and access control techniques will be used to ensure the protection of the patients’ data contained therein.

### Standards in interoperability and data security

European health systems have different ways of managing and storing health data, making the exchange of clinical data between the EU member states complex. The challenges are well illustrated by EHRs and their use as secondary research material. In a recent Organisation of Economic Collaboration and Development (OECD) report [22], ten countries reported comprehensive record sharing within one country-wide system designed to support each patient having only one EHR (Additional file 1). These countries are Estonia, Finland, France, Greece, Ireland, Latvia, Luxembourg, Poland, Slovakia, and the United Kingdom (England, Northern Ireland, Scotland, and Wales). In these countries, plans call for patient records regarding patient treatment, current medications, and laboratory tests and medical images to be shared among physician offices and between physicians and hospitals. Some have already implemented part or all these functionalities, while others are progressing toward it. In other countries, key aspects of record sharing are managed at sub-national level only, such as within provinces, states, regions, or networks of health care organizations (for example, Austria, Germany, Italy, Netherlands, Sweden, and Spain; Additional file 1). Among these countries, all have implemented or are planning the implementation of a national information exchange that enables key elements to be shared country-wide. On the basis of the recent reports of the European Commission [23], Belgium, Malta, Portugal, Romania, and Slovenia are now developing national EHR systems, leading to a total of 16 EU member states that will provide such services.

**Fig. 1:**
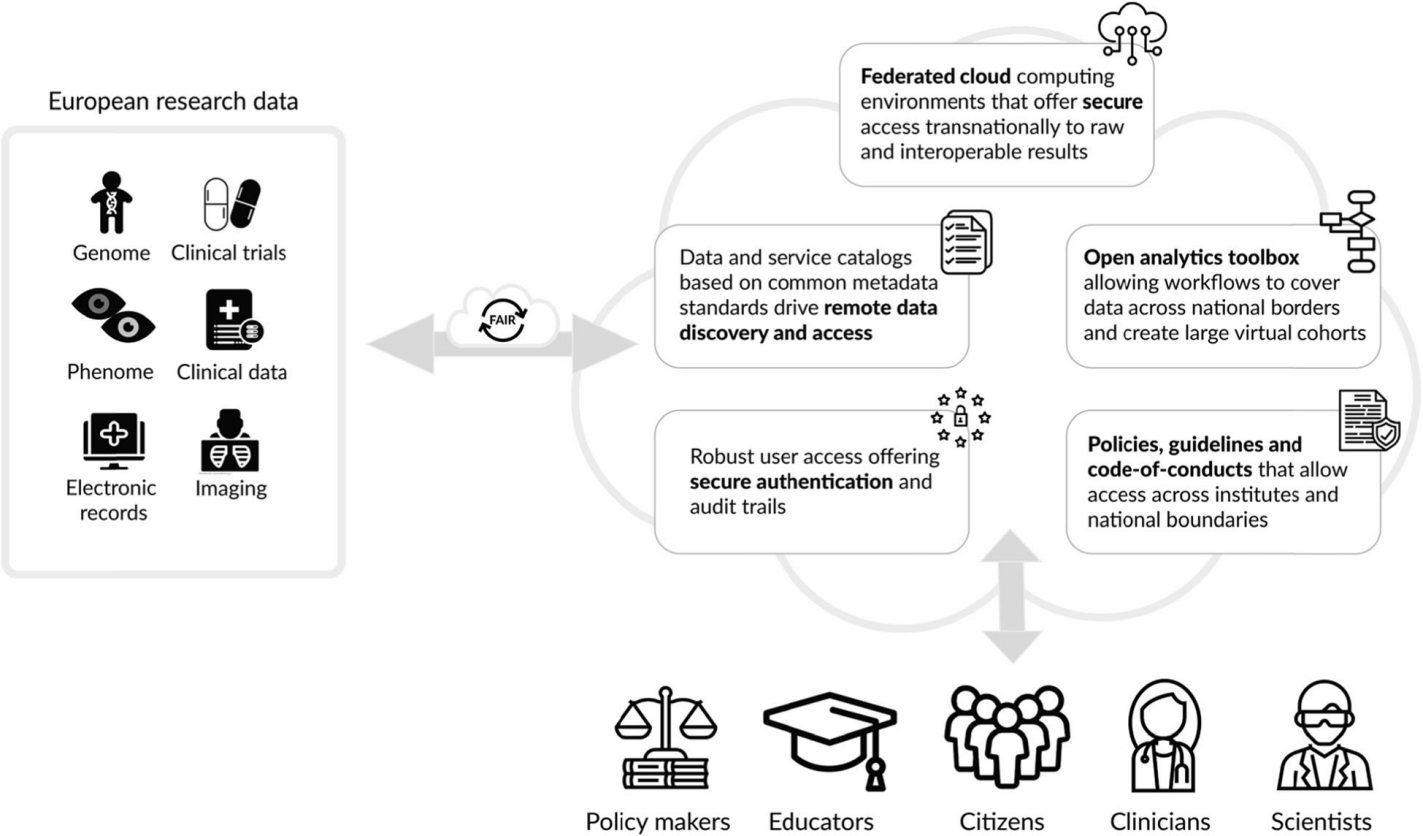
Proposed general architecture of the HRIC European (inter) national databases, with varying data formats and data types referenced in a metadata repository, following formatting rules of the federated data commons as agreed at the HRIC governance level. The different users, after access control to the cloud, use the HRIC interface to access the repository, which gathers the relevant data and performs analysis, with outputs such as mathematical models, data visualizations, statistics, and patient’s profiles according to the users’ needs

Within the framework of the Joint Action on Rare Cancers, an EU initiative that brings together European research centers, policy makers, and other stakeholders with the aim of setting the agenda at national level, an analysis was made on the status of eHealth medical records in the EU member states. This work builds on the OECD study and complements this with information provided by the European Commission on the national laws on EHRs in the EU member states [24]. Thus, all EU countries are investing in the development of clinical EHRs, but only some countries are moving forward the possibility of data extraction for research, the provision of statistics, and the enablement of other uses that serve the public interest (P. Bogaert, personal communication). Countries that develop EHR systems that combine or virtually link data together to capture patient health care histories can potentially use these for long-term follow-up of cancer patients. Figure 1 shows how data from various sources can be integrated to provide a full picture of patients’ health status over time and to carry out research on patterns and anomalies in large populations using the specific combinations of data and analysis resources relevant for each research project, while ensuring compliance with security and data protection regulations.

The H2020-funded project EOSC-Life [25] is developing policies, specifications, and tools for the management of data for biological and medical research, including aspects of eHealth data. The use of common metadata standards, developed in EOSC-Life, as a foundation for remote data discovery and access was emphasized by the workshop participants as a key enabler for the HRIC. For instance, practical and legal considerations for cloud computing of patient data, which include the responsible use of federated and hybrid clouds set up between academic and industrial partners, have been put forward by early EOSC pilot projects [26].

The workshop participants emphasized that sustainability aspects are critical and must be considered from the start. To ensure that a HRIC could respond properly to emerging needs, innovation, and technology changes, a distributed federated storage solution offering access to FAIR data and services should be built according to modularity principles. In particular, owing to the long-term nature of a HRIC, due consideration should be given to deploying generic and modular computational methods and/or data storage management systems, while the information and communications technology (ICT) infrastructure should be flexible, portable, and expandable.

The million European genomes initiative [27] is a case in point: 18 member states have already signed the Genomics Declaration of Cooperation [28] to enable cross-border access to genomic databases and other health information. This federation of national initiatives [28] will provide secure access to such data resources in the member states to enable the discovery of personalized therapies and diagnostics for the benefit of patients. The initiative involves aligning strategies of ongoing national genomic sequencing campaigns with complementary de novo genome sequencing to obtain a total cohort of one million Europeans, accessible in a transnational framework, by 2022 [29]. The HRIC would form a basis for such large-scale, permanent collaborations.

The workshop participants recognize that ensuring maximal data security is paramount in building and maintaining trust with European citizens. To address this issue, we suggest using modern cryptology such as blockchain to ensure data security by design, and holding regular data security assessments (for example, using hackathons and/or commercial security audits). As shown in recent literature, the use of blockchain in biomedical research is still in its infancy [30]. However, blockchain or other advanced cryptology tools that can be used to protect data in a cloud environment could prove useful in ensuring the secure and trustworthy implementation of the HRIC [31].

## The HRIC must be supported by a robust ethics and legal framework that is compliant with the general data protection regulation

### Rationale

Compliance with General Data Protection Regulation (GDPR) and other data protection laws, as well as enforcing an ethical usage of data, is paramount to gain the general public’s support and trust in the HRIC.

### Existing ethics and legal framework

Unblocking the legal and administrative barriers for sharing human research data across geographical and organizational boundaries will, if the trust of research participants is preserved, pave the way for continent-scale cohorts in life-science research. This will represent a significant innovation as the sharing and joint analysis of sensitive data has until now been severely limited because of the different restrictions inherent to the different classes of sensitive data. By using a federated database model, with a metadata repository within the HRIC cloud encrypted environment, data security is maintained while innovative data analyses can be performed by bringing the algorithms to the data rather than centralizing the data [32]. Federation, rather than full integration of all available resources, poses an important challenge for the implementation and deployment of an effective HRIC. The set-up and functioning of a HRIC requires a robust foundation of legal agreements and ethics rules and procedures, as well as security and data protection compliance protocols. Importantly, these elements must be introduced during the conception and design phase as part of HRIC governance. Indeed, in order to enable different HRIC actors to provide access to their data sources, manage these resources within the cloud, and access these resources, it is essential to incorporate policy requirements into the design of the HRIC itself and to manage the complexity involved by implementing simple and intuitive user interfaces and project portals. This may be challenging considering the heterogeneity of health systems and health market access across Europe and will need the sharing of a common agreed vision across EU member states.

Ethical, societal, and privacy considerations for (re) using health-related data have been outlined in the Code of Practice on Secondary Use of Medical Research Data, which was developed in the European Translational Information and Knowledge Management Services (eTRIKS) project funded by the Innovative Medicines Initiative (IMI) [33]. In addition to clear and explicit consent, explicit dissent may need to be considered for the use/re-use of data. Ultimately, each citizen and patient must be able to access her/his own data and know when and where it has been used and for what purpose. In addition, the difficult question of the business model of using those data should be discussed at various levels from ethics, social, and economics standpoints, taking into account the potential future development of products and services using personal medical data. Furthermore, the goal of providing citizens with personalized services requires technical advancements in the collection and analysis of data (for example, in data analytics and machine learning). For this type of usage, simple consent mechanisms might not be sufficient. For example, how should a clear data-collection purpose statement be defined if data are collected for multiple usage scenarios across a distributed/federated cloud, in which actors from different geographical and legislative environments will need to interact and cooperate? Would an excessive number of consent requests minimize data provision for research or clinical applications? Another level of complexity is introduced by the heterogeneity of data protection and privacy regulations when the data originate from states with federated national health systems (e.g., Germany and Italy). Development of large-scale European access mechanisms will require open consultation and engagement with national policymakers, patient organizations, and wider society to build the trust and confidence needed for widespread adoption and sustainable operations.

In addition to technical, ethical, and legal specifications, a global integrated governance model needs to be established for the HRIC that is in line with that of the EOSC, regulating the roles and responsibilities of all contributing institutions and users, and procedures for authentication and access control to individual resources. Principles, with specific guidelines on implementation in a HRIC environment, will need to be developed in order to manage and regulate aspects such as ownership, access, transparency, sharing, integration, standardization of data and metadata formats, tools, and frameworks, while ensuring confidentiality and sustainability. All of these principles need to be developed with the overarching objective of providing benefit to and preserving the trust of patients and the general public.

Health data mostly represent sensitive data, which need to be managed to preserve the trust of patients, research participants, and the general public, respect social norms, and naturally comply with the rules and regulations of data protection laws, notably the EU GDPR [15]. Although the GDPR directly applies across the EU and its provisions prevail over national laws, EU member states retain the ability to introduce their own national legislation under certain derogations provided for by the GDPR itself. The GDPR also introduces the notions of ‘Privacy by Design’, which means that any organization that processes personal data must ensure that privacy is built into a system during the whole life cycle of the system or process; and ‘Privacy by Default’, which means that the strictest privacy settings should apply by default, without any manual input from the end user. In addition, any personal data provided by the user to enable the optimal use of a given health dataset should only be kept for the amount of time necessary to provide the intended product or service [15].

Thus, successfully linking and accessing biomedical and health data across Europe will require many different disciplines and specialists working together, with a coordinated effort that should encompass controlled access mechanisms to ensure compliance with privacy and data protection regulations. Data providers need logging and monitoring functionalities to comply with the GDPR and to enable tracking of data and methods within the system, controlling instances and routines that check for the adherence to predefined standards and formats to guarantee data integrity. Access mechanisms need to be developed that support the researchers, data producers, and data analysts to request permissions and fulfill the reporting requirements for data use in national and international research projects; this is a significant regulatory, political, and sustainability challenge [34]. Such mechanisms include, in particular, considerations about the rights of patient donors and research participants, taking into account the data protection aspects of various legal systems and local regulations. Researchers have to face differences in the understanding of the right to data protection in those different regional or national European ecosystems.

There is an urgent need for standardized, usable, data-protection-policy-compliant solutions for sensitive data sharing which are capable of integrating and analyzing health data from different sources, organizations, and potentially from different research disciplines. These aspects are subject to ongoing discussions and debates in the EOSC initiative [35]; for instance, progress has been made in the Human Brain Project (HBP) through its Ethics and Society sub-project in collaboration with the project platforms [36, 37]. Other examples of data sharing that are compliant with data protection policies can be found in the recent literature [38–45]. Furthermore, there is the issue of capacity, with the amount of data starting to strain the infrastructure of any individual hospital or research institute. Thus, the interplay between privacy, data security, and access control on one hand and access (including cost-recovery models) to storage, computational, and analysis resources on the other hand will be a defining element of the policy and technology development of a decentralized digital health infrastructure. The evolution of a cloud model that could be used in European health research will also have to take into account other specific aspects of the GDPR [15]. For instance, the European Commission intends to facilitate the free flow of non-personal data in the European Digital Single Market, and for health-related research participants, it codifies the ‘right to be forgotten’. This stipulates that patient donors should be able to retain control over their data regardless of technological developments. A European HRIC could be important in enabling researchers to comply with these requirements. For example, once certain conditions are met between European and international partners, including those pertaining to data protection and use, federated and hybrid clouds could facilitate the deletion of data sets once a donor exercises her/his ‘right to be forgotten’, which could minimize the necessary transfer of large raw data sets across borders, as the deletion can be performed in the original dataset and easily propagated to the relevant federated data sources.

## A proper training environment for HRIC developers and users should be established

### Rationale

The workshop identified the lack of trained personnel, with solid skills in both medical and data analytical fields, as one of the major bottlenecks when dealing with ‘medical big data’ [3].

### Need for training and ideas

Effectively developing, operating, and maintaining the HRIC will pose serious challenges and will require the training of a new generation of data scientists who are able to navigate smoothly and efficiently between computational, security, and medical disciplines. This includes clinical researchers, bioinformaticians, data analysts, data managers, software engineers, cloud engineers, other IT-specialists, ethics officers, and data protection specialists, the latter representing an essential new field of expertise. Finding professionals who are able to cover more than one or two of the above-described disciplines is nearly impossible. Furthermore, communication between this comprehensive mix of clinical researchers, data managers, and IT/bioinformatics specialists needs to be improved, requiring a governance structure well beyond that of a standard research setting. The EU should take inspiration from existing large and successful infrastructures that foster multidisciplinary teams, such as the European Organisation for Nuclear Research (CERN) [46, 47]. Thus, it is necessary to rethink the training and education of health professionals and to update them with the HRIC in mind, considering both international standards and practices for data sharing, as well as national environments and regulations.

## Multiple funding mechanisms are required to drive the development of the HRIC and to support its broad use in research projects

### Rationale

Delivering the HRIC will require an ambitious reshaping of the European landscape for health data and research through appropriate funding schemes, which will enable the transformation of fragmented ICT resources and project-centric solutions for data access and governance into a long-term, coherent service ecosystem that can be accessed by users transnationally.

### Need for innovative public–private funding initiatives

The HRIC needs a trusted and transparent innovation approach that recognizes the importance of a clear, long-term ambition in the program to support the participation of industry and small-to-medium enterprises (SME) in joint projects with a broad set of societal actions. In particular, there is a need to support the EU ICT industrial/SME innovation ecosystem in order to demonstrate the benefits in advancing data sharing, integration, and analysis across Europe, for the benefit of all citizens, thus creating a foundation for attractive private investments.

For this purpose, targeted EU-funding mechanisms also involving private investors need to support the development of HRIC-compliant services for data sharing and analysis in health-related research projects (i.e., through reimbursement of storage and computing costs) with incentives to reuse and extend existing infrastructures that favor national HRIC participation rather than rebuilding and fragmenting solutions. Moreover, the EU ICT industrial ecosystem must be supported in order to alleviate the risks associated with storing and sharing data across cloud systems operated by non-EU companies. The EU has established a strict privacy and ethics policy through the implementation of the GDPR legislation, which is binding for all operators active in the EU territory [48–51].

European funders, science policy makers, and other actors need to develop mechanisms that bring together experience gained and lessons learned from a large portfolio of pathfinding projects and must build on current investments, thus leveraging existing project outcomes. This requires an inclusive and integrative approach with programs that bring together many different actors into the HRIC, because its construction needs interdisciplinary collaboration with expertise from many disciplines, including economics, ICT, biomedical and health, social sciences, and policy. In particular, frameworks for public–private partnerships such as IMI have shown a way to include industry in open transparent projects that also include patients and other public bodies, SMEs and European researchers. Many of the mechanisms proposed in the Lamy report (prioritize research and innovation in EU and national budgets, build a true EU innovation policy that creates future markets, rationalize the EU funding landscape and achieve synergy with structural funds …) [52] and in the development of the Horizon Europe ‘Missions’ [53] would also be well suited to the development of the HRIC, and would help to bring together initiatives from the many funders and national and regional stakeholders. Other opportunities, such as those proposed to be included in the Horizon Europe strategic workplan (e.g., the European Information Cloud, the European Institute of Technology, and the European Council for Health Research [54, 55]), should be of interest to those seeking to deploy innovations together with industry at the European level [56].

Furthermore, future programs need to create incentives such that developed solutions are transformed into long-term reusable resources and to make sure that this infrastructure is deployed across the EU with development informed by ongoing research projects. The European Joint Programme on Rare Diseases (EJP-RD) [56] provides a good example of how infrastructure development can be linked with research projects at national and international levels. Like the EJP-RD, the European Strategy Forum on Research Infrastructures (ESFRI) can play a role in the development of the HRIC. Two further aspects of the EJP-RD are worth noting: the program has a strong emphasis on the importance on the diverse workforce required, with a training program that stretches beyond academia and research networks to reach a broad set of individuals in health systems and the education sector. Moreover, the HRIC should fund as broadly as the EJP-RD and should recognize that successfully addressing many of the identified challenges will require a diverse portfolio of projects that avoid any artificial boundary between biomedical and health research. Horizon Europe should allow for linkages between the HRIC, ESFRI, and other themes of Horizon Europe and, importantly, between the HRIC and other funding sources such as the European Structural and Innovation Funds (ESIF) and the cutting-edge basic research successfully supported by the European Research Council (ERC) during the past decade [57, 58].

The HRIC should enable investments in product development for future health-care solutions and should allow health care providers to procure such solutions. People need to be an integral part of the innovation vision in which the HRIC supports a highly skilled future workforce that makes Europe attractive for locating R&D investments. The experience gained in IMI public–private partnership infrastructure projects such as eTRIKS and the European Medical Information Framework (EMIF) should be leveraged. Finally, the current and future EU Framework Programmes for Research and Innovation (Horizon 2020 and Horizon Europe) should consider mobilizing funds to support novel pilot actions and the pooling of data and resources across the EU, and should demonstrate the benefits in advancing data sharing, integration, and analysis across Europe, for the benefit of all citizens.

## Conclusions, recommendations, and action points

Clouds are increasingly becoming a key venue for enabling and hosting European and international collaborations, benefitting from the ability to hold data securely in a single location (or in few locations) and enabling collaborative research on the computational infrastructure used for analysis. In conclusion, a cloud-based federated data storage solution, with interoperable services for data-access to local repositories and modular environments that can be configured for a given use case, seems to match the data needs of the EU research and medical institutions and of all other stakeholders. The choice of cloud technology provides the ability to manage rapidly growing datasets and provides users with access to the massive computational infrastructure needed for analysis. The federated, cloud-based research environment described in this paper—HRIC—would represent an added value to the entire biomedical and bioinformatics community, because single research institutes and medical institutions lack sufficient infrastructure capacity. The establishment of a transnational HRIC will allow the European research community at large to contribute to the global international leadership required to address societal and scientific challenges through transnational collaborations. In order to ensure the effective and efficient implementation of the European HRIC, the workshop participants endorsed the five recommendations and action points presented in Table 2 to the EU and all stakeholders.

## Supplementary information

**Additional file 1.** National health record systems.

## Abbreviations

EHR: Electronic Health Records
EJP-RD: European Joint Program on Rare Diseases
EMR: Electronic Medical Records
EOSC: European Open Science Cloud
ESFRI: European Strategy Forum on Research Infrastructures
FAIR: Findable, Accessible, Interoperable, Reusable
FP7: The European Union’s seventh framework program for Research, Technological Development and Demonstration
GDPR: General Data Protection Regulation
HBP: Human Brain Project
HRIC: European Health Research and Innovation Cloud
ICT: Information and Communications Technology
IMI: Innovative Medicines Initiative
OECD: Organisation of Economic Collaboration and Development
SME: Small-to-Medium Enterprises
