# Supplementary material for: Towards a European health research and innovation cloud (HRIC)
Source: Genome Med. 2020 Feb 19;12:18. doi: 10.1186/s13073-020-0713-z (PMC7029532; doi:10.1186/s13073-020-0713-z)
Supplement: Supplementary file 1 — Additional file 1. National health record systems. [file 13073_2020_713_MOESM1_ESM.docx]

Additional file 1: National electronic health record systems

|  |  | Type of national system: | | | National system includes information sharing among: | | | | | | | | | | | | | | | |
| --- | --- | --- | --- | --- | --- | --- | --- | --- | --- | --- | --- | --- | --- | --- | --- | --- | --- | --- | --- | --- |
|  |  |  |  |  | Physician offices about: | | | | | | Physician offices and hospitals about: | | | | | | | | | |
| Country | Implementing a national electronic health record system | A country-wide electronic health record system (one system) | A system of exchange of patient data at the sub- national level only | Another type of system | Patient treatment | Current medications | | Laboratory tests | | Medical imaging results | | Patient treatment | Current medications | | Laboratory test results | | Medical imaging results | |  |  |
| Austria | Yes | Yes |  | Yes | Yes | | Yes | | Yes | Yes | | Yes | | Yes | | Yes | | Yes | |  |
| Belgium | Yes | Yes |  |  |  | |  | |  |  | |  | |  | |  | |  | |  |
| Croatia | No |  |  |  |  | |  | |  |  | |  | |  | |  | |  | |  |
| Cyprus | No |  |  |  |  | |  | |  |  | |  | |  | |  | |  | |  |
| Czech Republic | No |  |  |  |  | |  | |  |  | |  | |  | |  | |  | |  |
| Denmark | No |  |  | Yes |  | | Yes | | Yes | Yes | | Yes | | Yes | | Yes | | Yes | |  |
| Estonia | Yes | Yes |  |  | Yes | | Yes | |  | Yes | | Yes | | Yes | |  | | Yes | |  |
| Finland | Yes | Yes |  | Yes | Yes | | Yes | | Yes |  | | Yes | | Yes | | Yes | |  | |  |
| France | Yes | Yes |  |  | Yes | | Yes | | Yes | Yes | | Yes | | Yes | | Yes | | Yes | |  |
| Germany | Yes |  |  |  |  | |  | |  |  | |  | |  | |  | |  | |  |
| Greece | Yes | Yes |  |  | Yes | | Yes | |  |  | | Yes | | Yes | |  | |  | |  |
| Hungary | No |  |  |  |  | |  | |  |  | |  | |  | |  | |  | |  |
| Ireland | Yes | Yes |  | Yes |  | |  | |  |  | |  | |  | |  | |  | |  |
| Italy | Yes |  | Yes |  |  | |  | |  |  | |  | |  | |  | |  | |  |
| Latvia | Yes | Yes |  |  | Yes | | Yes | |  | Yes | | Yes | | Yes | |  | | Yes | |  |
| Lithuania | No |  |  |  |  | |  | |  |  | |  | |  | |  | |  | |  |
| Luxembourg | Yes | Yes |  | Yes | Yes | | Yes | | Yes | Yes | | Yes | | Yes | | Yes | | Yes | |  |
| Malta | Yes | Yes |  |  |  | |  | |  |  | |  | |  | |  | |  | |  |
| Netherlands | Yes |  | Yes |  |  | |  | |  |  | |  | |  | |  | |  | |  |
| Poland | Yes | Yes | Yes |  | Yes | | Yes | | Yes | Yes | | Yes | | Yes | | Yes | | Yes | |  |
| Portugal | Yes | Yes |  |  |  | |  | |  |  | |  | |  | |  | |  | |  |
| Romania | Yes | Yes |  |  |  | |  | |  |  | |  | |  | |  | |  | |  |
| Slovakia | Yes | Yes |  |  | Yes | | Yes | | Yes |  | | Yes | | Yes | | Yes | |  | |  |
| Slovenia | Yes (basic) |  |  |  |  | |  | |  |  | |  | |  | |  | |  | |  |
| Spain | Yes |  | Yes |  | Yes | | Yes | | Yes | Yes | | Yes | | Yes | | Yes | | Yes | |  |
| Sweden | Yes |  | Yes | Yes | Yes | | Yes | | Yes | Yes | | Yes | | Yes | | Yes | | Yes | |  |
| UK England | Yes | Yes |  |  | Yes | | Yes | | Yes | Yes | | Yes | | Yes | |  | | Yes | |  |
| UK Northern Ireland | Yes | Yes |  |  | Yes | | Yes | | Yes | Yes | | Yes | | Yes | | Yes | | Yes | |  |
| UK Scotland | Yes | Yes | Yes | Yes | Yes | | Yes | | Yes |  | |  | | Yes | | Yes | |  | |  |

Source: [[1](#_ENREF_1), [2](#_ENREF_2)]

1. **Overview of the national laws on electronic health records in the EU Member States and their interaction with the provision of cross-border eHealth services - Final report and recommendations** [https://ec.europa.eu/health/sites/health/files/ehealth/docs/laws_report_recommendations_en.pdf]

2. Oderkirk J: **Readiness of electronic health record systems to contribute to national health information and research**. In*.* Paris: OECD Health Working Papers; 217: 80.
